# Effects of task structure and confirmation bias in alternative hypotheses evaluation

**DOI:** 10.1186/s41235-024-00560-y

**Published:** 2024-06-13

**Authors:** Mandeep K. Dhami, Ian K. Belton, Peter De Werd, Velichka Hadzhieva, Lars Wicke

**Affiliations:** 1 Department of Psychology, Middlesex University, The Burroughs, Hendon, London, NW4 4BT UK; 2 University of Strathclyde, Glasgow, Scotland; 3 Netherlands Defence Academy, Breda, The Netherlands

**Keywords:** Hypothesis evaluation, Confirmation bias, Intelligence analysis, Cognitive reflection, Additivity

## Abstract

We empirically examined the effectiveness of how the Analysis of Competing Hypotheses (ACH) technique structures task information to help reduce confirmation bias (Study 1) and the portrayal of intelligence analysts as suffering from such bias (Study 2). Study 1 (*N* = 161) showed that individuals presented with hypotheses in rows and evidence items in columns were significantly less likely to demonstrate confirmation bias, whereas those presented with the ACH-style matrix (with hypotheses in columns and evidence items in rows) or a paragraph of text (listing the evidence for each hypothesis) were not less likely to demonstrate bias. The ACH-style matrix also did not confer any benefits regarding increasing sensitivity to evidence credibility. Study 2 showed that the majority of 62 Dutch military analysts did not suffer from confirmation bias and were sensitive to evidence credibility. Finally, neither judgmental coherence nor cognitive reflection differentiated between better or worse performers in the hypotheses evaluation tasks.

## Introduction

Intelligence analysts must search for, select, process, and interpret data in order to gain situational awareness and understanding of an issue or forecast an outcome of interest to policy-makers and decision-makers. Often, this requires analysts to assess evidence regarding alternative options. For example, analysts might consider alternative courses of action or responses by adversaries or other parties as part of the planning process for a military operation in light of a desired objective. Analysts may also consider the strength of a hostile state’s military in 5 years if it continues on its current trajectory versus under different economic scenarios due to sanctions. These so-called alternative hypotheses evaluation tasks are typically performed under suboptimal conditions. For instance, the availability and relevance of evidence may vary, as can the credibility of its source. In addition, analysts, like other people, have limited cognitive processing capacity that can affect their judgment. Perhaps unsurprisingly, critics have suggested that analysts may not apply a deliberative mode of thinking but instead resort to employing intuitive (simple or heuristic) cognitive strategies which can bias their judgment and result in errors (for a review see Belton & Dhami, 2021).

One common example of analytic bias is confirmation bias. According to psychological research (e.g., Klayman, 1995; Nickerson, 1998) confirmation bias may manifest in different ways. Although it is often considered that this bias refers to focusing on evidence that is consistent with (or confirms) an initially favored hypothesis while ignoring evidence inconsistent with (or disconfirms) it, the bias has other important elements. These include consideration of alternative hypotheses, adjusting belief in a hypothesis in accordance with evidence diagnosticity (or credibility), and identifying indicators that will disconfirm (or confirm) a hypothesis in the future.

Richards Heuer (1999), a Central Intelligence Agency (CIA) veteran and pioneering modernizer of analytic thinking, believed that analysts may reach conclusions about a hypothesis based primarily on the presence of supporting (confirming) evidence and may not sufficiently adjust their belief in a hypothesis when such evidence is discredited, and so they may select hypotheses that are in fact false. Heuer (1999) argued that techniques teaching deliberative (critical) thinking skills and boosting this mode of cognition can help analysts overcome bias (see also Heuer & Pherson, 2014). These suggestions closely followed the work of psychologists (Kahneman & Tversky, 1972; Tversky & Kahneman, 1971, 1974) who believe that intuitive thinking is the default mode of cognition which operates when the more superior, deliberative mode is unavailable (see also Kahneman, 2011; Kahneman & Klein, 2009). Therefore, many intelligence organizations nowadays provide training in critical thinking skills as well as encouraging their analysts to apply so-called structured analytic techniques (SATs, e.g., UK Ministry of Defence, 2013; US Government, 2009; for a review see Dhami et al., 2016).

The Analysis of Competing Hypotheses (ACH) is one commonly used SAT. It was designed by Heuer (1999, 2005) to overcome some elements of confirmation bias when performing alternative hypotheses evaluation. Specifically, ACH structures both the task information and the cognitive processes employed to perform the task. For present purposes, suffice it to say that ACH requires analysts to first prepare a matrix with hypotheses presented in columns and relevant evidence items for evaluating these hypotheses presented in rows (see Heuer & Pherson, 2014 for a more detailed account). Each evidence item is then considered as either being consistent or inconsistent with each hypothesis, taking account of evidence credibility. Finally, the relative likelihood of each hypothesis is judged by adding up the number of inconsistent scores for each hypothesis, so that the hypothesis with the fewest inconsistent scores is considered most likely whereas the one with the most inconsistent scores is judged to be least likely. ACH therefore attempts to encourage deliberative thinking by instructing analysts to weight (e.g., according to credibility) and integrate evidence inconsistent with a hypothesis, and attempts to help analysts avoid confirmation bias by instructing them to judge the likelihood of a hypothesis based on evidence inconsistent (rather than consistent) with it.

Heuer’s (1999, 2005) technique thereby effusively embraces the scientific principle of falsifiability proposed by Popper (1959). In addition, by presenting all task information in an ACH-style matrix, the technique directs evaluation of the alternative hypotheses by each evidence item in turn, thus focusing attention on the alternative hypotheses rather than the evidence. The matrix structure also ensures that all task information (i.e., hypotheses, evidence and interpretation of the evidence) remains cognitively available to the analyst (although some of it will eventually go unused). However, some argue that ACH misapplies the principle of falsification (Mandel, 2020). By focusing on disproving hypotheses, analysts may demonstrate ‘disconfirmation’ bias that can result in selecting a hypothesis which is in fact false. A deliberative strategy requires analysts to weight and integrate both confirming and disconfirming evidence. In addition, by encouraging analysts to evaluate alternative hypotheses by evidence item as per the matrix, ACH discourages analysts from taking account of dependencies between evidence items.

More broadly, critics of SATs such as ACH point out that these ‘debiasing’ interventions are not informed by the relevant psychological evidence base and that their effectiveness is rarely rigorously tested (e.g., Belton & Dhami, 2021; Chang et al., 2018; de Melo, 2021; Dhami et al., 2015; Mandel, 2020). Chang and Tetlock (2016) further note that SATs could potentially lead analysts to over-compensate for a bias thereby resulting in an opposing bias (e.g., over v. under-confidence). In addition, some have argued that the prevalence of cognitive bias in intelligence analysis is overstated (e.g., Dhami & Careless, 2019; Dhami et al., 2019; Klein, 2010).

## Relevant past research on analyst thinking, bias and ACH

There is as yet only a small body of research on analytic thinking, bias and SATs such as ACH. Much of the extant research is based on extremely small samples of analysts and/or non-analysts (e.g., Chin et al., 2009; Convertino et al., 2008; Dhami & Careless, 2015; Kretz & Granderson, 2013; Kretz et al., 2012; Lehner et al., 2008; Patterson et al., 2001; Pirolli & Card, 2005; Roth et al., 2010; Tolcott et al., 1989; Trent et al., 2007). However, some recent studies have employed larger samples and yielded insightful observations (Dhami & Careless, 2019; Dhami et al., 2019; Maegherman et al., 2020; Mandel et al., 2018).

Dhami and Careless (2019) surveyed 113 analysts, asking them how often they would apply specific strategies when performing representative tasks along each stage of the analytic workflow. Unbeknownst to the analysts, these strategies had been a priori labeled as either ‘deliberative’ or ‘intuitive’ (e.g., involved little effort) by analytic trainers and managers. It was found that analysts reported using deliberative strategies significantly more often than intuitive ones when performing tasks along three stages of the workflow (i.e., capturing customer requirements, processing data, and communicating conclusions) and were equally likely to report applying deliberative and intuitive strategies at the other three stages (i.e., plan analytic response, obtain data and interpret outputs). Thus, analysts may not typically suffer from the sorts of cognitive biases that arise from intuitive thinking. In addition, there was little association between strategy use and analysts’ experience, skill level and training, suggesting that the findings were not moderated by these potentially relevant variables.

Dhami, Mandel and their colleagues conducted a randomized controlled trial of ACH where half of a sample of 50 analysts were trained (and instructed) to use ACH (experimental group) and half were not (control group), before all completed an alternative hypotheses evaluation task involving four hypotheses and 12 evidence items (Dhami et al., 2019; Mandel et al., 2018). The researchers collected written protocol data from analysts as they solved the task, as well as quantitative survey data, after they solved the task. An examination of the in-task data (Dhami et al., 2019) revealed that 80% of the control group reformatted the textual data provided to them into an ACH-style matrix. Most analysts in the ACH group departed from ACH’s suggestion that only evidence inconsistent with a hypothesis should be used to judge the likelihood of a hypothesis. Analysts in the ACH group who provided at least one indicator for future observation, were equally likely to provide potentially confirming and disconfirming indicators. Across both groups, only a small minority of analysts added up solely evidence consistent with each hypothesis, with the majority adding up both consistent and inconsistent evidence. Finally, analysts in the ACH group were less likely to demonstrate internal consistency in their judgment processes compared to those in the control group, and they did not demonstrate greater judgmental accuracy. Examination of the post-task data (Mandel et al., 2018) provided further support for these findings. It indicated that ACH may actually reduce the logical coherence of probability judgments, and may increase judgment error both in terms of mean absolute error across judgments of the probability of each hypothesis and in terms of the rank order of these hypotheses.

Finally, Maegherman et al. (2020) also tested ACH using a randomized controlled trial, although in the legal context. Law students (*N* = 191) read a scenario indicating that a suspect may be guilty of an offence and were given an opportunity to collect evidence which could either further incriminate the suspect or potentially lead to an exoneration. It was found that participants in both the ACH group (who were trained in the ACH procedure) and the control group (who simply received information on cognitive bias) chose significantly more questions that could disconfirm than confirm the suspect’s guilt. Both groups rated the exonerating evidence as significantly more important than the incriminating evidence. There was also no difference between the groups in terms of their ratings of the likelihood of the suspect’s guilt either before or after further evidence collection, with both groups rating the suspect’s guilt as significantly less after evidence collection. Finally, only around a third of the ACH group reported using the ACH matrix, with the average ‘helpfulness’ of the matrix being rated as 57% (out of 100 meaning ‘very helpful’).

## The present research

In the present paper, we present two studies contributing to the extant literature on analytic thinking, bias and SATs. In Study 1, we experimentally examine the effect of how information is structured (i.e., an ACH-style matrix, a different matrix, or paragraph of text) on the strategies that analysts use to evaluate alternative hypotheses. To our knowledge, no research has yet examined the effect of an ACH-style matrix where hypotheses are presented in columns and evidence items in rows, despite the stated belief that such a matrix would improve analytic judgment (e.g., Davies & Gustafson, 2017; Heuer, 2005). In Study 2, we involve analysts from a different population to those studied previously (i.e., Dutch military) in order to re-examine the assumption that analysts demonstrate confirmation bias when evaluating alternative hypotheses. It is important to note that we are not directly testing the effectiveness of ACH. Instead, we are investigating a specific feature of ACH (Study 1) and we are reassessing the portrayal of analyst cognitive behavior as suffering from confirmation bias that has given rise to ACH (Study 2). As mentioned earlier, confirmation bias can manifest in several ways. We examined the bias in terms of reaching conclusions about a hypothesis based solely on the presence of supporting (consistent) evidence, and resisting change or insufficiently adjusting confidence in a hypothesis when existing supporting evidence is discredited.

The understanding gleaned from addressing these issues can be used to reconsider the resources invested in training analysts to use existing SATs such as ACH and can contribute to the development of new, psychologically evidence-based interventions (see also Belton & Dhami, 2021). Such research is timely in light of continued recommendations for the use of SATs to solve intelligence problems (e.g., Coulthart, 2017; Davies & Gustafson, 2017; Hart, 2014; Lemay & Leblanc, 2018; Stromer-Galley et al., 2021). In addition, the proliferation of SATs beyond the national intelligence domain to law enforcement and the business intelligence sector (e.g., Houck, 2020; Townsley et al., 2011; see also Maegherman et al., 2020) reinforces the need to ensure that analytic policies and practices in these other domains benefit from the emerging psychological evidence base.

## Study 1^1^

As mentioned, in addition to structuring the cognitive process that analysts employ to evaluate alternative hypotheses, ACH also structures the task information. All hypotheses are presented in columns and evidence items in rows of a matrix, with the (dis)confirmatory nature of the evidence specified in the hypothesis-by-evidence item cells. It is unknown how this structure reduces confirmation bias and/or confers other benefits such as increasing sensitivity to evidence credibility relative to other potential ways of structuring task information. In fact, the ACH-style matrix may not be the most helpful or effective way of structuring task information. By presenting hypotheses in columns and evidence in rows, ACH encourages evaluation of alternative hypotheses evidence item by item, and since the task is to determine which hypothesis is most likely, this presentation requires simultaneous processing of information about each hypothesis. Thus, at minimum, a deliberative approach to evaluating alternative hypotheses using the ACH-style matrix would require the capacity to remember whether or not an item of evidence is consistent or inconsistent with each hypothesis before integrating this information per hypothesis and comparing the outputs. This is cognitively demanding, and the challenge is greater when one considers that evidence items may not be applicable to one of the hypotheses (and so need to be discarded), and given the additional burden of assessing, weighting and integrating the credibility of evidence items. By contrast, a structure that facilitates *sequential* processing of information about each hypothesis may make the task less cognitively demanding, thus affording greater capacity to apply deliberative thinking when evaluating alternative hypotheses.

In addition, since the number of alternative hypotheses to be evaluated is typically much smaller than the number of evidence items, viewing information in an ACH-style matrix does not capitalize on the physiology of human binocular vision, which includes a wider field of vision and stronger muscles in the horizontal dimension (Deng et al., 2016; Ojanpää et al., 2002). Information arranged vertically, as in an ACH-style matrix, may therefore be more difficult to process than information arranged horizontally. Indeed, the dominance of horizontal over vertical eye movements has been observed in many eye-tracking studies (e.g., Gilchrist & Harvey, 2006; Shi et al., 2013) and working in a horizontal direction has been linked to greater self-reported perceptual fluency (Deng et al., 2016). Finally, most cultures (especially in the Western world) read and write horizontally (often using left-to-right eye movements). Consequently, a structure that entails viewing information *horizontally* may better facilitate information processing when evaluating alternative hypotheses. The main aim of Study 1 was to compare the effect of an ACH-style matrix against other ways of structuring task information on the strategies that individuals use to evaluate alternative hypotheses.

A secondary aim of Study 1 was to explore the relationship between individuals’ evaluation of alternative hypotheses and their judgmental coherence and cognitive reflection. Some have argued that intelligence organizations ought to recruit and select the ‘right kind’ of individuals (e.g., Dhami & Mandel, 2021; Karvetski et al., 2013; Mellers et al., 2015a, 2015b). In order to be coherent, judgments of the likelihood of (mutually exclusive) hypotheses ought to sum to unity, but some individuals have been shown to be nonadditive; either demonstrating superadditivity (sum to less than unity) or subadditivity (sum to more than unity; Rottenstreich & Tversky, 1997; Tversky & Koehler, 1994; for nonadditivity in analyst samples see Mandel, 2015; Mandel et al., 2018). Similarly, some individuals appear to have greater ability to reflect on a decision problem and refrain from providing the first response that comes to mind while others have less ability to do so (Frederick, 2005; see also Campitelli & Gerrans, 2014; Pennycook et al., 2016). Both judgmental coherence and cognitive reflection have been shown to be positively related to performance on a range of judgment and decision-making tasks (e.g., Baron et al., 2015; Campitelli & Labollita, 2010; Fan et al., 2019; Frederick, 2005; Karvetski et al., 2013; Mandel et al., 2018; Mellers et al., 2015a, 2015b; Moritz et al., 2013; Pajala, 2019; Toplak et al., 2011). These two individual difference measures may therefore be useful in analyst selection.

### Method

#### Design

Information structure was manipulated in a between-subjects experimental design. There were three levels of information structure: an ACH-style matrix (which we call HypCol), a matrix with hypotheses in rows and evidence items in columns (HypRow), and text listing the evidence for each hypothesis in turn (HypText).

#### Participants

One hundred and sixty-one staff and students from a UK university volunteered to participate in return for a £10 gift voucher. For our main analyses, a power analysis using G*Power 3 (Faul et al., 2007) for a chi-square test with 2 degrees of freedom, to obtain 0.90 power to detect a medium-sized effect (0.3) with an alpha of 0.05 requires a sample of 141. The average age of the sample was 20.02 (*SD* = 3.53, min = 17, max = 42), 90.1% were female, and 85.1% said the highest level of education they had reached to-date was ‘A level’ (i.e., educated up to 18 years). Twenty-three percent said they had been trained to use the scientific method of experimental design, and this knowledge was not associated with condition, *p* = 0.865.

#### Stimuli

Each participant completed three alternative hypotheses evaluation tasks from a set of four previously designed by Belton and Dhami (2016). All tasks involve two hypotheses and 12 evidence items. Given that avoiding confirmation bias is the primary goal of ACH, in the present study we focused on distinguishing between individuals who rely solely on evidence consistent with a hypothesis (hereafter called CONS strategy) and those who rely either solely on evidence inconsistent with a hypothesis (INCONS strategy) or those who balance both types of evidence (BAL strategy).^2^

All four tasks developed by Belton and Dhami (2016) are presented in Appendix A (using an ACH-style matrix) along with a detailed explanation of how their design helps to discriminate among strategies or establish sensitivity to evidence credibility. In the present study we used Tasks 1, 3A and 3B. Task 1 was used to discriminate among use of the CONS strategy and the INCONS or BAL strategy. Here, as detailed in Appendix A, use of a CONS strategy would result in choosing Hypothesis-A, whereas use of either a BAL or INCONS strategy would result in choosing Hypothesis-B. This discrimination is possible because of the number of evidence items in the task that were said to be consistent and/or inconsistent with each hypothesis (see ‘Appendix 1’).

Tasks 3A and 3B were used to establish individuals’ sensitivity to evidence credibility. As detailed in Appendix A, this is possible because of the way credibility levels (i.e., high, medium, low) were assigned to evidence items that were consistent and/or inconsistent with each hypothesis. Here, those who choose Hypothesis-A in Task 3A and then switch to Hypothesis-B in Task 3B are classified as being sensitive to evidence credibility.

In the HypCol condition (i.e., the ACH-style matrix), the information was presented in a matrix with hypotheses in columns and evidence items in rows. In the HypRow condition, the information was presented in a matrix with hypotheses in rows and evidence items in columns (see ‘Appendix 2’). Finally, in the HypText condition, the information was presented as a paragraph of text for each hypothesis listing the evidence items in relation to that hypothesis (see ‘Appendix 2’).

#### Measures

For each task, all participants responded to a set of questions. First, they judged the likelihood of each hypothesis being true on a 0–100% scale (with 5% intervals). These responses were used to measure individual differences in additivity in judgments.

Then, participants chose which hypothesis they believed was most likely to be true (i.e., A or B). As mentioned above, these choices helped to establish participants’ strategy use and sensitivity to evidence credibility (see also ‘Appendix 1’). Specifically, participants choosing Hypothesis-A in Task 1 are classified as using a CONS strategy, whereas those choosing Hypothesis-B are classified as using either a BAL or INCONS strategy. Participants who switch their hypothesis choice from Hypothesis-A in Task 3A to Hypothesis-B in Task 3B are considered to be sensitive to evidence credibility.

Participants were also asked to describe how they reached their conclusion in an open-ended response. Following this, participants rated the usefulness of each evidence category, namely consistent (C), highly consistent (CC), inconsistent (I), highly inconsistent (II), and not applicable (NA). Ratings were provided on 11-point scales anchored at each end from 0 (‘not at all useful’) to 10 (‘extremely useful’). These open-ended responses and ratings were used to further validate classification of participants’ strategy use.

After completing the three alternative hypotheses evaluation tasks, participants were asked to recall how the evidence in the tasks was presented to them (i.e., sentences, a matrix with hypotheses in rows, or a matrix with hypotheses in columns). This was to determine if they were aware of structural aspects of the task.

Finally, in order to measure individual differences in cognitive reflection participants completed the Cognitive Reflection Test (CRT, Frederick, 2005).^3^ The CRT comprises three questions: (1) A bat and a ball cost $1.10 in total. The bat costs $1.00 more than the ball. How much does the ball cost? (2) If it takes 5 machines 5 min to make 5 widgets, how long would it take 100 machines to make 100 widgets? (3) In a lake, there is a patch of lily pads. Every day the patch doubles in size. If it takes 48 days for the patch to cover the entire lake, how long would it take for the patch to cover half of the lake? Each correct answer scores one point, producing a total score of between zero and three.

#### Procedure

The present study received ethics approval from Middlesex University, Department of Psychology Research Ethics Committee. Data were collected in the Psychology Department laboratory, in small groups. Participants were randomly assigned to one of the three conditions (HypCol: *n* = 55, HypRow: *n* = 51, Text: *n* = 55). Data collection comprised an individual, self-completion, paper–pencil procedure. There was no time limit for completion of the tasks. Participants first completed the tasks (see ‘Appendix 3’ for task instructions), followed by some demographic questions.

### Results

#### Strategy use

Recall that Task 1 distinguished between individuals who used a CONS strategy (i.e., choosing Hypothesis-A) and those using either a BAL or INCONS strategy (i.e., choosing Hypothesis-B; see ‘Appendix 1’). Across conditions, 56.5% selected Hypothesis-A, and so were classified as using a CONS strategy, with the remainder (43.5%) who selected Hypothesis-B, being classified as using a BAL or INCONS strategy.

Individuals’ strategy use, based on hypothesis choice, was compatible with their self-reported strategy use. Specifically, self-reported strategy use was coded blind (i.e., without knowledge of their hypothesis choice and their experimental condition) into three categories (i.e., relying solely on hypothesis-consistent evidence, relying solely on inconsistent evidence, and relying on both categories of evidence).^4^ There was a statistically significant association with self-reported strategy use and hypothesis choice in Task 1, *χ*^2^(2) = 28.85, *p* < 0.001, *φ* = 0.49. Overall, 74.6% of those who chose Hypothesis-A (indicative of a CONS strategy) were also more likely to report relying solely on consistent evidence compared to 25.4% of those who chose Hypothesis-B (indicative of a BAL or INCONS strategy).

Individuals’ strategy use (based on their hypothesis choice) was also compatible with their ratings of the usefulness of different categories of evidence (i.e., C and CC v. I and II). After averaging individuals’ ratings of how useful they found each evidence category (i.e., C and CC versus I and II) in Task 1, we found that across conditions, those who selected Hypothesis-A (indicative of using a CONS strategy) were significantly more likely to rate evidence category C/CC as more useful (*M* = 5.64, *SD* = 2.27) than evidence category I/II (*M* = 5.05, *SD* = 2.23), *t*(90) = 2.43, *p* = 0.008, *d* = 0.26.^5^

#### Effect of information structure on strategy use^6^

Table 1 shows the percentage and number of individuals in each condition who chose Hypothesis-A and Hypothesis-B, respectively, in Task 1. As can be seen, the majority of the HypRow group chose Hypothesis-B (indicative of either a BAL/INCONS strategy), whereas the majority of the HypCol and HypText groups chose Hypothesis-A (indicative of a CONS strategy). There was a statistically significant association between hypothesis choice (i.e., strategy use) and information structure, *χ*^2^(2) = 9.43, *p* = 0.009, *φ* = 0.24. Post hoc analyses with Bonferroni corrections^7^ revealed that the HypRow group was significantly less likely to choose Hypothesis-A than B which is indicative of a CONS strategy, *p* = 0.003. By contrast, there was no significant difference in hypothesis choice for the HypCol group (*p* = 0.327) and hypothesis choice only approached ‘borderline’ significance for the HypText group (*p* = 0.048).

**Table 1:** Hypothesis choice in Task 1 by information structure condition

Condition	Hypothesis-A % (*n*)	Hypothesis-B % (*n*)
HypCol	61.8 (34)	38.2 (21)
HypRow	39.2 (20)	60.8 (31)
HypText	67.3 (37)	32.7 (18)

#### Sensitivity to evidence credibility

Individuals who chose Hypothesis-A in Task 3A and then switched to Hypothesis-B in Task 3B were classified as being sensitive to evidence credibility. In order to establish this sensitivity therefore, individuals must have chosen Hypothesis-A in Task 3A. However, 18%, 15% and 35% of those in the HypCol, HypRow and HypText conditions, respectively, chose Hypothesis-B. Excluding them, 77% of individuals in the HypCol switched to Hypothesis-B in Task 3B, thus demonstrating sensitivity to evidence credibility. Similarly, 78% switched in the HypRow condition and 75% did so in the HypText condition. Finally, there was no significant association between information structure and sensitivity to evidence credibility, *χ*^2^(2) = 0.15, *p* = 0.927, *φ* = 0.04.

#### Judgmental coherence, cognitive reflection and alternative hypotheses evaluation

On average, individuals’ CRT score was 0.51 (SD = 0.87).^8^ As Table 2 shows, the majority of individuals in each condition were nonadditive (across tasks), and of these, most were subadditive (i.e., their likelihood judgments of each Hypothesis being true summed to greater than unity). There was also no significant correlation between CRT score and mean absolute deviation from additivity across tasks, for each condition (see Table 2).

**Table 2:** Means and standard deviations of the absolute deviation from additivity (across tasks) by condition and correlations with CRT score

Condition	Mean absolute deviation from additivity across tasks (%)		Correlation with CRT score	Additivity of participants (%)		
	*M*	*SD*	*r*	Super	Add	Sub
HypCol	16.17	11.17	− .07	41.8	1.8	56.4
HypRow	20.01	16.23	− .11	39.2	3.9	56.9
HypText	16.77	13.04	.02	41.8	7.3	50.9

Mean absolute deviation from additivity across tasks and conditions = 17.59 (SD = 13.59)

Next, we explored the relationship between the two individual difference measures and strategy use as well as sensitivity to evidence credibility, taking account of information structure. A one-way analysis of variance found that CRT score did not differ significantly across information structures, *F*(2, 60) = 2.30, *p* = 0.103, *η*^2^ = 0.03. Similarly, there was no significant relationship between information structure and mean absolute deviation from additivity across tasks *F*(2,160) = 1.22, *p* = 0.299, *η*^2^ = 0.02. Thus, unless otherwise stated the remainder of the analyses is conducted across conditions.

We found a significant relationship between CRT score and strategy use. Specifically, across conditions, those individuals who chose Hypothesis-A in Task 1 (i.e., indicative of a CONS strategy) scored significantly lower on the CRT (*M* = 0.27, *SD* = 0.68) than those who chose Hypothesis-B (indicative of either a BAL or INCONS strategy; *M* = 0.81, *SD* = 1.00), *t*(116) = 3.88, *p* < 0.001, Glass’s *d* = 0.54.^9^ However, across conditions, there was no significant difference in absolute deviation from additivity in Task 1 and individuals’ hypothesis choice (strategy use) in Task 1, *t*(159) = 0.25, *p* = 0.803, *d* = 0.04)^10^ suggesting that absolute deviation from additivity was unrelated to strategy use.

Logistic regression analyses with mean absolute deviation from additivity across tasks, CRT score, and information structure (included to test for interactions with the other predictors) as predictor variables and sensitivity to evidence credibility as the outcome variable, found that neither individual difference measure significantly predicted sensitivity to evidence credibility, and there were no interactions between information structure and the other two predictors, all *p*s > 0.082.

### Discussion

In Study 1, we compared the effects of information structured in an ACH-style matrix (HypCol) against two other ways of structuring information, i.e., a matrix with hypotheses in rows and evidence items in columns (HypRow) and a paragraph of text for each hypothesis listing the evidence items in relation to that hypothesis (HypText). Individuals performing an alternative hypotheses evaluation task where hypotheses were presented in rows and evidence items in columns were significantly less likely to show confirmation bias (i.e., rely solely on the presence of evidence consistent with a hypothesis). This was in contrast to those presented with information in either an ACH-style matrix or as a paragraph of text who were not less likely to show confirmation bias. In addition, the ACH-style matrix did not confer any benefits over the other two ways of structuring task information in terms of increasing individuals’ sensitivity to evidence credibility.

Thus, the findings of Study 1 do not support the belief that an ACH-style matrix would improve analytic judgment by eliminating confirmation bias (e.g., Davies & Gustafson, 2017; Heuer, 2005). The ACH-style matrix (HypCol) directs evaluation of the alternative hypotheses by each evidence item in turn, thus focusing attention on the alternative hypotheses rather than the evidence, whereas the HypRow format directs attention to the evidence, better enabling individuals to consider evidence in terms of (in)consistency. The present findings are arguably compatible with Maegherman et al.’s (2020) observation that law students trained to use ACH gave an average rating of only 57% regarding the helpfulness of the ACH-style matrix, and only around a third of students used the matrix. The ACH-style matrix may therefore not be the most helpful or effective way of structuring information in an alternative hypotheses evaluation task, despite analysts’ ‘natural’ proclivity to reformat textual information in this way (Dhami et al., 2019).

Presenting hypotheses in rows and evidence items in columns (i.e., HypRow) places the alternative hypotheses evaluation task on a horizontal dimension. This capitalizes on the physiology of human binocular vision, is compatible with the convention of reading on a horizontal plane and fosters the less cognitively demanding sequential processing of information about each hypothesis. However, further research is needed to replicate and extend the present findings. Specifically, future research should vary the numbers of alternative hypotheses and evidence items. Future research should also establish the effect of the HypRow structure on individuals’ use of a BAL strategy that integrates both consistent and inconsistent evidence as opposed to an INCONS strategy that relies solely on inconsistent evidence, given that in Study 1 we could not distinguish between these two strategies. Finally, future research ought to examine how different ways of structuring the task information affects the evaluation of alternative hypotheses performed collaboratively (as opposed to individually as in Study 1). Heuer (2005, p. 93) argues that ‘ACH is also an excellent framework for collaboration between analysts. The cross-fertilization of ideas helps analysts generate more and better ideas. The matrix can combine inputs from analysts with different specialties. When analysts disagree, the matrix can be used to highlight the precise area of disagreement.’

A secondary aim of Study 1 was to explore the relationship between analysts’ evaluation of alternative hypotheses and their judgmental coherence and cognitive reflection. Although judgmental coherence was unrelated to strategy use, we found that individuals higher in cognitive reflection were less likely to rely solely on the presence of evidence consistent with a hypothesis. Others have previously found cognitive reflection to be positively related to performance on a range of judgment and decision-making tasks (e.g., Baron et al., 2015; Campitelli & Labollita, 2010; Frederick, 2005; Mellers et al., 2015a; Moritz et al., 2013; Pajala, 2019; Toplak et al., 2011). The CRT may therefore be a useful tool for analyst selection, in addition to the other individual difference measures such as intelligence, open-minded thinking and numeracy that have been previously suggested (e.g., Karvetski et al., 2013; Mellers et al., 2015a, 2015b). However, it is necessary to further explore the positive relationship between CRT and strategy use identified in Study 1 for two reasons. First, meta-analytic findings suggest that CRT scores tend to be lower for females (who made up the majority of participants in Study 1; Brañas-Garza et al., 2019), and so it is unclear if the findings generalize to analyst samples (who are more likely to be male). Second, in Study 1 we were unable to explore the relationship between CRT scores and use of a BAL and INCONS strategies separately, whereas in Study 2 we will distinguish between them and so can conduct this analysis.

In fact, the idea that we need different kinds of analysts is partly based on research suggesting that people lack abilities relevant to specific sorts of analytic tasks, namely forecasting (Karvetski et al., 2013; Mellers et al., 2015a, 2015b). These studies were either based on non-analyst samples or a mix of analysts and lay people, and none examined analysts’ ability to evaluate alternative hypotheses. In Study 2, we therefore re-examine analysts’ ability to evaluate alternative hypotheses employing a sample from a different population to those studied previously (e.g., Dhami et al., 2019; Mandel et al., 2018).

## Study 2^11^

The negative characterization of analysts that has given rise to the use of SATs such as ACH is not necessarily warranted by recent evidence. As mentioned earlier, Dhami and Careless’s (2019) survey of UK analysts found no predominance of intuitive strategy use either across their sample of analysts or within specific sub-groups (e.g., those with less experience, skill level and training). Furthermore, Dhami et al.’s (2019) and Mandel et al.’s (2018) analyses of experimental data on ACH showed little evidence of confirmation bias in UK analysts. Rather, these studies demonstrated that analysts may apply a deliberative or more cognitively sophisticated strategy than that dictated by ACH. In addition, research has demonstrated that analysts are relatively good at other sorts of analytic tasks such as strategic forecasting (Mandel & Barnes, 2018). The primary aim of Study 2 was therefore to reassess the assumption that analysts (untrained in ACH) will demonstrate confirmation bias when evaluating alternative hypotheses. We also determined if analysts are sensitive to evidence credibility. Study 2 builds on previous research by involving analysts from a different population to that studied previously (i.e., Dutch military).

A secondary aim of Study 2 was to further explore the relationship between individual differences in judgmental coherence and cognitive reflection and performance on an alternative hypotheses evaluation task (measured in terms of strategy use and sensitivity to evidence credibility).

### Method^12^

#### Participants

Sixty-two Dutch intelligence analysts and officer cadets attending training at the Defence Intelligence and Security Institute in the Netherlands or the Defence Academy volunteered to participate in the study without reimbursement.^13^ Participation was anonymous. Eighty percent of the sample was male, and the average age was 32.48 years (*SD* = 8.99, min = 18, max = 54).

#### Stimuli

In the present study, each analyst completed all four of the alternative hypotheses evaluation tasks shown in ‘Appendix 1,’ and these were presented in the ACH-style matrix (i.e., what was called HypCol in Study 1). Two tasks (i.e., Tasks 1 and 2) discriminate among three strategies, namely a strategy that relies solely on the presence of evidence consistent with a hypothesis (CONS strategy), a strategy that relies solely on the presence of evidence inconsistent with a hypothesis (INCONS strategy), and a strategy that balances both types of evidence (BAL strategy). As detailed in ‘Appendix 1,’ those who choose Hypothesis-A in Task 1 and Hypothesis-B in Task 2 can be classed as using a CONS strategy. Those who choose Hypothesis-B in Task 1 and Hypothesis-A in Task 2 can be classed as using an INCONS strategy, and those who choose Hypothesis-B in both tasks can be classed as using a BAL strategy. This discrimination is possible because of the number of evidence items in the tasks that were said to be consistent and/or inconsistent with each hypothesis. Finally, Tasks 3A and 3B were used to establish individuals’ sensitivity to evidence credibility. Here, as in Study 1, those who choose Hypothesis-A in Task 3A and then switch to Hypothesis-B in Task 3B are classified as being sensitive to evidence credibility.

#### Measures

For each task, all analysts first judged the likelihood of each hypothesis being true on a 0–100% scale (with 5% intervals) and then chose which hypothesis they believed was most likely to be true (i.e., A or B). Analysts also completed the CRT, among other items.^14^

#### Procedure

The present study received ethics approval from Middlesex University, London, Department of Psychology Research Ethics Committee. Data were collected by the third author on the first day of training using an individual, self-completion, paper–pencil procedure at the defense training facilities. Analysts first completed the four alternative hypotheses evaluation tasks (see ‘Appendix 3’ for task instructions), followed by the CRT and some demographic questions. There was no time limit for completion of the tasks.

### Results

#### Strategy use

Analysts were first classified as either using a CONS, INCONS or BAL strategy based on their pattern of hypothesis choice across both Tasks 1 and 2.^15^ Those who chose Hypothesis-A in Task 1 and Hypothesis-B in Task 2 were classified as using a CONS strategy. Those who chose Hypothesis-B in Task 1 and Hypothesis-A in Task 2 were classified as using an INCONS strategy, and those who chose Hypothesis-B in both tasks were classified as using a BAL strategy. Overall, 19.4% of analysts were classified as using a CONS strategy, 14.5% as using an INCONS strategy and 66.1% as using a BAL strategy.

#### Sensitivity to evidence credibility

Next, analysts’ sensitivity to evidence credibility was established based on their pattern of hypothesis choice across Tasks 3A and 3B.^16^ Those who chose Hypothesis-A in Task 3A and then switched to Hypothesis-B in Task 3B were classified as being sensitive to evidence credibility. All but one participant chose Hypothesis-A in Task 3A. Excluding the exception, 83.3% of analysts switched to Hypothesis-B in Task 3B, thus demonstrating sensitivity to evidence credibility.

#### Relationship between strategy use and sensitivity to evidence credibility

The relationship between strategy use and sensitivity to evidence credibility was also examined. Ninety-eight percent of analysts classified as using a BAL strategy were sensitive to evidence credibility, compared to 72.7% using a CONS strategy and 33.3% using an INCONS strategy. Logistic regression analyses revealed that strategy use was a significant predictor of sensitivity to evidence credibility (see Table 3). Specifically, analysts classified as using a CONS strategy or INCONS strategy were both less likely to be sensitive to evidence credibility compared to those classified as using a BAL strategy.

**Table 3:** Effect of strategy on sensitivity to evidence credibility, as measured by likelihood of switching from Hypothesis-A to Hypothesis-B

Predictor	*b*	S.E	Exp(*b*)	95% C.I. Exp(*b*)		*p*
				Lower	Upper	
Constant	3.66	1.01	39.00			< .001
Strategy						
CONS	− 2.68	1.22*	.07	.01	.74	.028
INCONS	− 4.36	1.24***	.013	.00	.14	< .001

Strategy was a three-level categorical variable, with BAL being the reference category

#### Judgmental coherence, cognitive reflection and alternative hypotheses evaluation

On average, analysts’ CRT score was 2.32 (*SD* = 0.92). The mean absolute deviation from additivity across tasks was 15.42 (*SD* = 12.23). The majority (91.9%) of analysts were nonadditive; 24.2% were superadditive and 67.7% were subadditive (i.e., their likelihood judgments summed to greater than unity). There was no significant correlation between analysts’ CRT score and their mean absolute deviation from additivity across tasks, *r* = − 0.12, *p* = 0.374. In what follows, we explore the relationship between these two individual difference measures and analysts’ strategy use as well as their sensitivity to evidence credibility, including analyses comparable to that in Study 1.

A one-way analysis of variance found no significant relationship between strategy use (i.e., CONS, INCONS and BAL) and CRT score, *F*(2, 61) = 1.30, *p* = 0.279, *η*^2^ = 0.04. In analysis comparable to Study 1 (where strategy use was defined as CONS v. BAL/INCONS), we again found no significant difference in CRT score of those classified as using a CONS strategy (*M* = 2.25, *SD* = 0.75) and those classified as using either a BAL or INCONS strategy (*M* = 2.34, *SD* = 0.96), *t*(60) = 0.30, *p* = 0.763, *d* = 0.10.

We examined the association between absolute deviation from additivity and strategy use in two different ways. First, in analysis comparable to Study 1, we focused on the hypothesis choice in Task 1 (which distinguished between analysts using a CONS strategy versus a BALS/INCONS strategy) and absolute deviation from additivity in Task 1. As in Study 1, there was no significant difference between the two groups in terms of absolute deviation from additivity, *t*(60) = 0.39, *p* = 0.699, *d* = 0.13. Second, we compared analysts classified as using a BAL strategy with those using either a CONS or INCONS strategy. This distinguishes between analysts who used some sort of heuristic strategy versus those who were more cognitively complex. There was no significant difference between these two groups in terms of their absolute deviation from additivity in either Task 1 or Task 2, *t*(60) = 0.69, *p* = 0.494, *d* = 0.19 and *t*(60) = 0.63, *p* = 0.530, *d* = 0.17, respectively.

Finally, a logistic regression analysis found that neither CRT score nor mean absolute deviation from additivity across tasks were significant predictors of whether or not analysts were sensitive to evidence credibility, all *p*s > 0.143.

### Discussion

In Study 2, we re-examined the assumption that analysts demonstrate confirmation bias when evaluating alternative hypotheses. We found that instead of relying solely on evidence consistent with a hypothesis (i.e., a demonstration of confirmation bias), most analysts integrated evidence for and against each hypothesis (i.e., BAL strategy) and were sensitive to evidence credibility. Indeed, strategy use was a significant predictor of sensitivity to evidence credibility, with those analysts who either relied solely on evidence consistent (i.e., CONS strategy) or inconsistent with a hypothesis (i.e., INCONS strategy; as ACH mandates) being less sensitive to evidence credibility compared to those classified as using a BAL strategy. The present findings, based on Dutch analysts, are compatible with recent research involving UK analysts, who also wanted to apply a more cognitively sophisticated strategy than that dictated by ACH (Dhami et al., 2019; Mandel et al., 2018). More generally, the behavior observed in Study 2 is compatible with other recent research on UK analysts which revealed their use of a deliberative (rather than intuitive) mode of thinking (Dhami & Careless, 2019). Together, these findings paint a different picture of analyst cognitive behavior than that which has given rise to the use of SATs such as ACH.

Nevertheless, we are not saying that all analysts are deliberative when performing all analytic tasks. Rather, we argue that it is important to empirically examine analyst behavior in order to determine the sorts of support and guidance they may need. In addition, it may be useful to determine whether some individuals need more support and guidance than others. It is, for example, argued that beyond not being impulsive or using intuitive strategies which may result in cognitive bias and error, deliberative thinking also requires individuals to be internally coherent in their judgments (e.g., Rottenstreich & Tversky, 1997; Tversky & Koehler, 1994). Indeed, only a minority of analysts were additive in their judgments of the likelihood of each hypothesis being true, and the mean absolute deviation from additivity across all tasks was not significantly different from that displayed by non-analysts in Study 2, *t*(221) = 1.10, *p* = 0.136, *d* = 0.16. Around two-thirds of analysts in Study 2 demonstrated subadditivity (i.e., their likelihood judgments summed to more than unity), and although other samples of analysts also demonstrate nonadditivity, they have to-date been mostly characterized as superadditive (i.e., their judgments sum to less than unity; Mandel, 2015; Mandel et al., 2018). The pattern of behavior observed in Study 2 is similar to that found in Study 1 and is compatible with other research on non-analyst samples (Dhami & Mandel, 2013; Rottenstreich & Tversky, 1997; Wallsten et al., 1993). Also, as in Study 1, we did not find a significant association between judgmental coherence and strategy use or sensitivity to evidence credibility. The importance of the role of judgmental coherence in hypothesis choice is unclear, because only 1.6% of analysts in Task 2 and 4.8% in Task 1 selected the hypothesis they judged to be least likely, and this individual difference measure may not be particularly useful in determining whether or not some individuals need more support and guidance when performing alternative hypotheses evaluation tasks.

Study 2 also confirmed the observation from Study 1 of a lack of relationship between CRT score and additivity. To our knowledge, the present research is the first to measure the relationship between judgmental coherence and cognitive reflection. In addition, Study 2 confirmed the observation from Study 1 of a lack of relationship between CRT score and sensitivity to evidence credibility. This is notable because the analysts in Study 2 (*M* = 2.32, *SD* = 0.92) scored significantly higher on the CRT than did the non-analyst sample in Study 1 (*M* = 0.51, SD = 0.87),* t*(221) = 13.68, *p* < 0.001, *d* = 2.05. In fact, analysts scored higher on the CRT relative to other samples more generally (see Brañas-Garza et al., 2019). This suggests that analysts may be largely able to reflect on a decision problem and refrain from providing the first response that comes to mind (Frederick, 2005; see also Campitelli & Gerrans, 2014; Pennycook et al., 2016). However, contrary to Study 1, we did not find a significant relationship between CRT score and strategy use (either when comparing a CONS v. BAL/INCONS strategy as in Study 1, or when comparing a BAL v. CONS/INCONS strategy). Further research is clearly warranted before we can recommend using the CRT as a tool for analyst selection.

## General discussion

Many intelligence organizations train their analysts to employ SATs (for a review see Dhami et al., 2016), and there is particular enthusiasm for ACH, which aims to help analysts overcome confirmation bias when evaluating alternative hypotheses (UK Ministry of Defence, 2013; US Government, 2009). Recent efforts to improve analytic performance involve embedding elements of ACH, without questioning its utility, in a web-based application that affords analysts some flexibility (Stromer-Galley et al., 2021). Indeed, SATs such as ACH are rarely empirically evaluated and/or evidence-based. Rather, they are developed and accepted based on their face validity, with the belief that ‘something is better than nothing.’

The present research empirically tested a specific feature of ACH, namely how it structures task information in a matrix (Study 1) and reassessed the portrayal of analysts as suffering from confirmation bias when evaluating alternative hypotheses (Study 2). Furthermore, both studies explored the potential utility of individual differences in judgmental coherence and cognitive reflection in differentiating among performers in alternative hypotheses evaluation tasks. Study 1 suggests that the ACH-style matrix may not be the most helpful or effective way of structuring task information, and rather structuring information in the opposing way (i.e., hypotheses in rows and evidence items in columns) may be more beneficial. Study 2 provides further evidence that analysts may use a deliberative (rather than intuitive) mode of thinking when evaluating alternative hypotheses (i.e., they integrate both evidence consistent and inconsistent with each hypothesis rather than rely solely on evidence consistent with a hypothesis). Finally, both studies suggest that judgmental coherence may not be useful for differentiating among performance in alternative hypotheses evaluation tasks. Similarly, the usefulness of the cognitive reflection measure may be limited.

Although we did not directly test the effectiveness of ACH as others have done (e.g., Dhami et al., 2019; Kretz & Granderson, 2013; Kretz et al., 2012; Lehner et al., 2008; Maegherman et al., 2020; Mandel et al., 2018), the understanding gleaned from addressing the issues in the present research can be used to reconsider the resources invested in training analysts to use existing SATs such as ACH, developing new, evidence-based interventions, and identifying useful tools for analyst selection. While the present research itself cannot provide definitive guidance on these issues, it does demonstrate the potential utility of a psychological evidence-based approach to such policy and practice. Below, we point to directions for future research which continue in this endeavor, and highlight the main strengths and limitations of our approach.

### Strengths, limitations and future research directions

The alternative hypotheses evaluation task used in the present research was purposefully designed to discriminate among different strategies (and establish sensitivity to evidence credibility). This is akin to approaches used in other research aiming to identify individuals’ judgment strategy use (e.g., Garcia-Retamero & Dhami, 2009; Rieskamp & Otto, 2006). However, it could be argued that we have determined individuals’ strategy use on the basis of a few tasks (i.e., one in Study 1 and two in Study 2). We believe that our approach is preferable to the reliance on self-reports or post hoc analysis of behavioral data, which typically also involve one or few tasks. The fact that the task has been previously pilot-tested (Belton & Dhami, 2016), and that the classification of participants in the present research was consistent with other data (i.e., self-reported strategy use and ratings of usefulness of different evidence categories) lends the classification some reliability and validity.

However, we do concede that the present findings are based on tasks involving two hypotheses and 12 evidence items, and so it would be prudent to examine analysts’ performance on other configurations of hypotheses and evidence items. Such research could also examine how analysts take account of dependencies between evidence items, given that, as mentioned earlier, the ACH-style matrix discourages this through its emphasis on evaluating alternative hypotheses by evidence item.

Additionally, the tasks we used capture specific manifestations of confirmation bias, namely reaching conclusions about a hypothesis based solely on the presence of supporting evidence, and resisting change or insufficiently adjusting confidence in a hypothesis when existing supporting evidence is discredited. Although ACH itself also does not directly tackle all elements of confirmation bias identified in the psychological literature (see Klayman, 1995; Nickerson, 1998), future research could nevertheless examine other manifestations of this bias. These include whether analysts remain overconfident in an initial position, whether they search for and/or interpret new evidence in a way that favors an existing hypothesis, and whether they are resistant to change in response to new conflicting evidence. Research in the law enforcement domain suggests that individuals may demonstrate confirmation bias in the form of drawing on evidence that favors an initial position, and this bias is reduced when individuals actively consider alternative scenarios (O’Brien, 2009; Rassin, 2018). Similarly, early research has shown that a ‘consider-the-opposite’ strategy can reduce the tendency to discount conflicting evidence (Lord et al., 1984).

In sum, a psychologically evidence-based approach could lead to more effective policies and practices in intelligence analysis, that could ultimately reduce the likelihood of biases and errors, while also increasing accountability processes (Dhami et al., 2015). Empirically evaluating SATs such as ACH is timely in light of continued recommendations for their use to solve intelligence problems (e.g., Coulthart, 2017; Davies & Gustafson, 2017; Hart, 2014; Lemay & Leblanc, 2018; Stromer-Galley et al., 2021). The fact that there is a proliferation of SATs such as ACH beyond the national intelligence domain to other domains involving analytic work such as in the legal and criminal justice system (e.g., Houck, 2020; Townsley et al., 2011) also means that research akin to that presented here is potentially widely applicable.

## Data Availability

The datasets generated and/or analyzed during the present research are available in the Open Science Framework repository at https://osf.io/hcdp3/.

## Appendix 1

### Alternative hypotheses evaluation task set (taken from Belton & Dhami, 2016)

All tasks involve two hypotheses and 12 evidence items.

### Tasks discriminating among strategies

Task 1 (see below) first distinguishes between individuals who used a strategy that relies solely on the presence of evidence consistent with a hypothesis (hereafter called CONS strategy) and individuals who used either a strategy that relies solely on the presence of evidence inconsistent with a hypothesis (INCONS strategy) or a strategy that balances both types of evidence (BAL strategy).

Task 2 (see below) then distinguishes between individuals who used an INCONS strategy and those who used either a CONS or BAL strategy. (It is assumed that evidence regarded as CC or II was given twice the weight of evidence considered as C or I, respectively.)

In Task 1, the evidence consistent with Hypothesis-A is greater (i.e., 4 CCs + 1 C = 9) than Hypothesis-B (i.e., 1 CC + 4 Cs = 6). The evidence inconsistent with Hypothesis-A is also greater (i.e., 3 IIs + 4 Is = 10) than Hypothesis-B (i.e., 4 Is = 4). The remaining evidence items are said to be Not Applicable (NA) for each hypothesis. Thus, choosing Hypothesis-A suggests the use of a CONS strategy, whereas favoring Hypothesis-B suggests the use of either an INCONS strategy or BAL strategy (i.e., for Hypothesis-A: 9 − 10 = − 1; Hypothesis-B: 6 − 4 = 2).

In Task 2, the evidence consistent with Hypothesis-B is greater (i.e., 4 CCs + 2 Cs = 10) than Hypothesis-A (i.e., 4 Cs = 4). The evidence inconsistent with Hypothesis-B is also greater (i.e., 2 IIs + 3 Is = 7) than Hypothesis-A (i.e., 4 Is = 4). The remaining evidence items are not applicable (NA) for each hypothesis. Here, the choice of Hypothesis-A suggests the use of an INCONS strategy, whereas favoring Hypothesis-B suggests use of either a CONS strategy or BAL strategy (i.e., for Hypothesis-A: 4 − 4 = 0; Hypothesis-B: 10 − 7 = 3).

Taken together, individuals who choose Hypothesis-A in Task 1 and Hypothesis-B in Task 2 can be classed as using a CONS strategy. Those who choose Hypothesis-B in Task 1 and Hypothesis-A in Task 2 can be classed as using an INCONS strategy, and those who choose Hypothesis-B in both tasks can be classed as using a BAL strategy.

Task 1

**Table:**

	Hypothesis-A	Hypothesis-B
Evidence from source 1	CC	I
Evidence from source 2	II	C
Evidence from source 3	I	C
Evidence from source 4	CC	I
Evidence from source 5	I	NA
Evidence from source 6	CC	I
Evidence from source 7	C	I
Evidence from source 8	I	NA
Evidence from source 9	II	CC
Evidence from source 10	CC	C
Evidence from source 11	I	C
Evidence from source 12	II	NA

Task 2

**Table:**

	Hypothesis-A	Hypothesis-B
Evidence from source 1	NA	CC
Evidence from source 2	C	NA
Evidence from source 3	NA	I
Evidence from source 4	I	CC
Evidence from source 5	NA	I
Evidence from source 6	I	C
Evidence from source 7	C	II
Evidence from source 8	C	CC
Evidence from source 9	NA	I
Evidence from source 10	I	II
Evidence from source 11	C	C
Evidence from source 12	I	CC

### Tasks establishing sensitivity to evidence credibility

Task 3A (see below) first establishes individuals’ hypothesis choice before information on evidence credibility is provided. Task 3B (see below) then establishes if participants switched their hypothesis choice after this information. Ideally, all individuals (regardless of strategy use) will initially choose the same Hypothesis-And then only switch to the alternative hypothesis if they are sensitive to information about evidence credibility. Evidence credibility is described as ‘high,’ ‘medium’ or ‘low’ following Heuer and Pherson (2014). Importantly, the switch to the other hypothesis occurs under different assumptions about how the levels of evidence credibility might be used. Specifically, low credibility evidence may be either ignored or given a lesser weight than the other two levels of evidence credibility (i.e., low = 0 or low = 1 if medium = 2 and high = 3), and high credibility evidence may be given an equal weight as medium credibility evidence (i.e., high = 1 and medium = 1) or double the weight as medium credibility evidence (i.e., high = 2 and medium = 1).

In Task 3A the evidence consistent with Hypothesis-A is greater than Hypothesis-B. In addition, the evidence inconsistent with Hypothesis-B is greater than Hypothesis-A. The remaining evidence items are NA for each hypothesis. Thus, in Task 3A all strategies (i.e., CONS, INCONS and BAL) favor Hypothesis-A.

In Task 3B, four of the 12 evidence items are regarded as low credibility, four as medium credibility, and four as high credibility. All of the evidence consistent with Hypothesis-A is of low or medium credibility, and all the evidence inconsistent with it is of medium or high credibility. All of the evidence consistent with Hypothesis-B is of medium or high credibility, and all but one evidence item inconsistent with it is of low or medium credibility (and the one exception was of high credibility). Thus, individuals who are sensitive to information on evidence credibility would choose Hypothesis-B in Task 3B.

Taken together, we would expect sensitive individuals to switch from Hypothesis-A in Task 3A to Hypothesis-B in Task 3B.

Task 3A

**Table:**

	Hypothesis-A	Hypothesis-B
Evidence from source 1	NA	C
Evidence from source 2	C	NA
Evidence from source 3	C	I
Evidence from source 4	I	C
Evidence from source 5	CC	II
Evidence from source 6	I	C
Evidence from source 7	CC	II
Evidence from source 8	I	CC
Evidence from source 9	NA	I
Evidence from source 10	I	C
Evidence from source 11	CC	I
Evidence from source 12	C	NA

Task 3B

**Table:**

	Credibility	Hypothesis-A	Hypothesis-B
Evidence from source 1	High	NA	C
Evidence from source 2	Low	C	NA
Evidence from source 3	Medium	C	I
Evidence from source 4	Medium	I	C
Evidence from source 5	Low	CC	II
Evidence from source 6	High	I	C
Evidence from source 7	Low	CC	II
Evidence from source 8	High	I	CC
Evidence from source 9	High	NA	I
Evidence from source 10	Medium	I	C
Evidence from source 11	Low	CC	I
Evidence from source 12	Medium	C	NA

## Appendix 2

### Study 1, task 1 and 3 materials for HypRow and HypText conditions (see ‘Appendix 1’ for HypCol condition)

Task 1—*HypRow Condition.*

**Table:**

	Evidence source 1	Evidence source 2	Evidence source 3	Evidence source 4	Evidence source 5	Evidence source 6	Evidence source 7	Evidence source 8	Evidence source 9	Evidence source 10	Evidence source 11	Evidence source 12
Hypothesis-A	CC	II	I	CC	I	CC	C	I	II	CC	I	II
Hypothesis-B	I	C	CC	I	NA	I	I	NA	CC	C	C	I

Task 1—*HypText Condition*

Hypothesis-A is highly consistent with evidence from source 1; highly inconsistent with evidence from source 2; inconsistent with evidence from source 3; highly consistent with evidence from source 4; inconsistent with evidence from source 5; highly consistent with evidence from source 6; consistent with evidence from source 7; inconsistent with evidence from source 8; highly inconsistent with evidence from source 9; highly consistent with evidence from source 10; inconsistent with evidence from source 11; and highly inconsistent with evidence from source 12.

Hypothesis-B is inconsistent with evidence from source 1; consistent with evidence from source 2; highly consistent with evidence from source 3; inconsistent with evidence from source 3; not applicable to evidence from source 5; inconsistent with evidence from source 6; inconsistent with evidence from source 7; not applicable to evidence from source 8; highly consistent with evidence from source 9; consistent with evidence from source 10; consistent with evidence from source 11; and inconsistent with evidence from source 12.

Task 3—*HypRow Condition*

**Table:**

	Evidence source 1	Evidence source 2	Evidence source 3	Evidence source 4	Evidence source 5	Evidence source 6	Evidence source 7	Evidence source 8	Evidence source 9	Evidence source 10	Evidence source 11	Evidence source 12
Credibility	High	Low	Medium	Medium	Low	High	Low	High	High	Medium	Low	Medium
Hypothesis-A	NA	C	C	I	CC	I	CC	I	NA	I	CC	C
Hypothesis-B	C	NA	I	C	II	C	II	CC	I	C	I	NA

Task 3—*HypText Condition*

Source 1 has high credibility. Source 2 has low credibility. Source 3 has medium credibility. Source 4 has medium credibility. Source 5 has low credibility. Source 6 has high credibility. Source 7 has low credibility. Source 8 has high credibility. Source 9 has high credibility. Source 10 has medium credibility. Source 11 has low credibility. Source 12 has medium credibility.

Hypothesis-A is highly consistent with evidence from source 1; highly inconsistent with evidence from source 2; inconsistent with evidence from source 3; highly consistent with evidence from source 4; inconsistent with evidence from source 5; highly consistent with evidence from source 6; consistent with evidence from source 7; inconsistent with evidence from source 8; highly inconsistent with evidence from source 9; highly consistent with evidence from source 10; inconsistent with evidence from source 11; and highly inconsistent with evidence from source 12.

Hypothesis-B is inconsistent with evidence from source 1; consistent with evidence from source 2; highly consistent with evidence from source 3; inconsistent with evidence from source 3; not applicable to evidence from source 5; inconsistent with evidence from source 6; inconsistent with evidence from source 7; not applicable to evidence from source 8; highly consistent with evidence from source 9; consistent with evidence from source 10; consistent with evidence from source 11; and inconsistent with evidence from source 12.

## Appendix 3: Instructions to stimuli

### Study 1

The background information to Tasks 1, 2 and 3 was as follows: ‘Imagine that you are a senior intelligence analyst. A junior analyst has assessed how a set of evidence from various different sources relates to two competing *hypotheses* (i.e., possible explanations for a currently unexplained event or a predicted future event). There are 12 evidence items in total, each from a different source.’

In the HypCol and HypRow conditions, the instructions continued: ‘The junior analyst has prepared a matrix that indicates whether the evidence is *highly consistent* (CC), *consistent* (C), *inconsistent* (I) or *highly inconsistent* (II) with each hypothesis, or *not applicable* to that hypothesis (NA). Your task is to evaluate the two hypotheses based on the information in the matrix. Please assess the evidence in the matrix and decide which hypothesis is most likely to be true.’

In the HypText condition, there were no further instructions.

There were additional instructions for Task 3: ‘Task 3 is the same as Task 2 but with some additional information provided about the **credibility** of the different sources. The credibility of each source is described as either *high, medium* or *low*. Please weight the evidence based on its credibility and review your decision accordingly.’

### Study 2

The background information to Tasks 1, 2 and 3A was as follows: ‘Intelligence analysts are required to assess evidence to test alternative accounts of a current situation or a future one. We want you to imagine that another analyst has assessed how a set of evidence from various different sources relates to two competing hypotheses. The analyst has prepared a matrix that indicates whether the evidence is *highly consistent* (CC), *consistent* (C), *inconsistent* (I) or *highly inconsistent* (II) with each hypothesis, or whether it is *not applicable* (NA). Your task is to evaluate the two hypotheses based on the information in the matrix, and answer the questions that follow.’

There were additional instructions for Task 4 which said ‘Matrix 3B is the same as Matrix 3A but with some additional information provided about the credibility of the different sources. Please answer the questions that follow.’

## Abbreviations

ACH: Analysis of Competing Hypotheses
CIA: Central Intelligence Agency
SATs: Structured analytic techniques
HypCol: ACH-style matrix
HypRow: Matrix with hypotheses in rows and evidence items in columns
HypText: Text listing the evidence for each hypothesis in turn
CONS strategy: Rely solely on evidence consistent with a hypothesis
INCONS strategy: Rely solely on evidence inconsistent with a hypothesis
BAL strategy: Balance both evidence consistent and inconsistent with a hypothesis
C: Consistent
CC: Highly consistent
I: Inconsistent
II: Highly inconsistent
NA: Not applicable
CRT: Cognitive Reflection Test
UK: United Kingdom
US: United States of America

## Footnotes

1 This study was not preregistered.

2 Distinguishing between the latter two strategies would have required using all four tasks which would place additional time burden on participants while reducing the financial reimbursement’s appeal.

3 Participants also completed other items making up the Actively Open-Minded Thinking scale, which has subsequently been shown to lack internal validity (Janssen et al., 2020) and so is not used here.

4 Thirty-five individuals’ open-ended responses could not be reliably coded, and two did not provide a response.

5 One-tailed test.

6 The relationship between condition and individuals’ accuracy in recalling how the task information was presented to them was not statistically significant, χ^2^(2) = 4.19, *p* = .123, φ = .16. The majority of those in each condition demonstrated accurate recall (HypCol: 76.4%, HypRow: 74.5%, HypText: 60.0%).

7 The adjusted residuals for each cell in the 3 × 2 contingency table were converted into chi-square values, and their statistical significance was established following adjustment to the alpha-level.

8 Although the test can be scored in different ways, the scoring approach has been shown to be unrelated to other indicators of cognitive style such as ‘need for cognition’ (Erceg & Bubić, 2017), and so we employ the original  scoring rule of summing the correct answers (Frederick, 2005).

9 For unequal variances.

10 This finding also holds when examining the mean absolute deviation from additivity across tasks and hypothesis choice on Task 1 which is indicative of strategy use, *t*(169) = 0.43, *p* = .669, *d* = .07.

11 This study was not preregistered.

12 This was one of a set of six separate studies that the analysts participated in. The studies were divided into two separate blocks and this study was the second of block one, with the preceding study being on an unrelated topic.

13 Determination of sample size was outside the control of the authors because access was granted to a limited number of training sessions.

14 As in Study 1, the other items were a subset of the Actively Open-Minded Thinking scale which we do not use here due to its lack of internal validity. In addition, analysts responded to a larger set of individual difference measures that were relevant for the other (unrelated) studies conducted with this sample.

15 Both hypotheses were judged to be equally likely by five participants for Task 1 and three for Task 2. For the remainder of participants, with three exceptions for Task 1 and two for Task 2, all chose the hypothesis they judged to be most likely.

16 Both hypotheses were judged to be equally likely by three participants for Matrix 3A and one for Matrix 3B. For the remainder of participants, all chose the hypothesis they judged to be most likely for Matrix 3A, and for Matrix 3B there were two exceptions to this.
